# Bilateral Transposition Flaps With Split-Thickness Skin Grafting of Secondary Defects After a Large Mohs Micrographic Surgery Defect With Exposed Calvarium

**DOI:** 10.7759/cureus.42191

**Published:** 2023-07-20

**Authors:** S. Caleb Freeman, Brett Neill, Christopher Garvey, Justin J Leitenberger

**Affiliations:** 1 Dermatology, Oregon Health & Science University, Portland, USA; 2 Dermatology and Mohs Micrographic Surgery, Swann Dermatology Partners, Springfield, USA; 3 Skin Cancer Treatment, Cosmetic Dermatology, General Dermatology, Epiphany Dermatology, Rockwall, USA

**Keywords:** large scalp defect, dermatology surgery, split-thickness skin graft, transposition flap, mohs micrographic surgery, dermatology

## Abstract

Large, full-thickness defects of the scalp create a common reconstructive dilemma following Mohs micrographic surgery. In cases with exposed calvarium, transposition flap(s) followed by split-thickness skin graft(s) to the secondary defect is an effective method of reconstruction that allows for same-day repair, full defect coverage, and good functional outcomes. Herein, we present the reconstruction of a large scalp defect utilizing bilateral transposition flaps followed by split-thickness skin grafts of the secondary defects.

## Introduction

Surgical treatment of cutaneous malignancies on the scalp can result in a wide variety of defects requiring unique approaches to reconstruction. Deeply extending tumors can be particularly challenging, especially when treatment results in a defect with exposed bone. Skin grafts are unlikely to survive over-exposed calvarium due to lack of necessary perfusion, which is typically provided by the overlying periosteum. With very large defects, even single or multiple rotation flaps do not provide adequate defect coverage and are frequently avoided due to poor laxity of scalp tissue complicating the ability to repair secondary defects. Here, we report the reconstructive dilemma of a large scalp defect measuring 7.5 x 6.0 cm, devoid of periosteum, which was repaired utilizing bilateral transposition flaps to the primary defect followed by split-thickness skin (STSG) grafts to repair the secondary defect sites. 

## Case presentation

**Figure 1:**
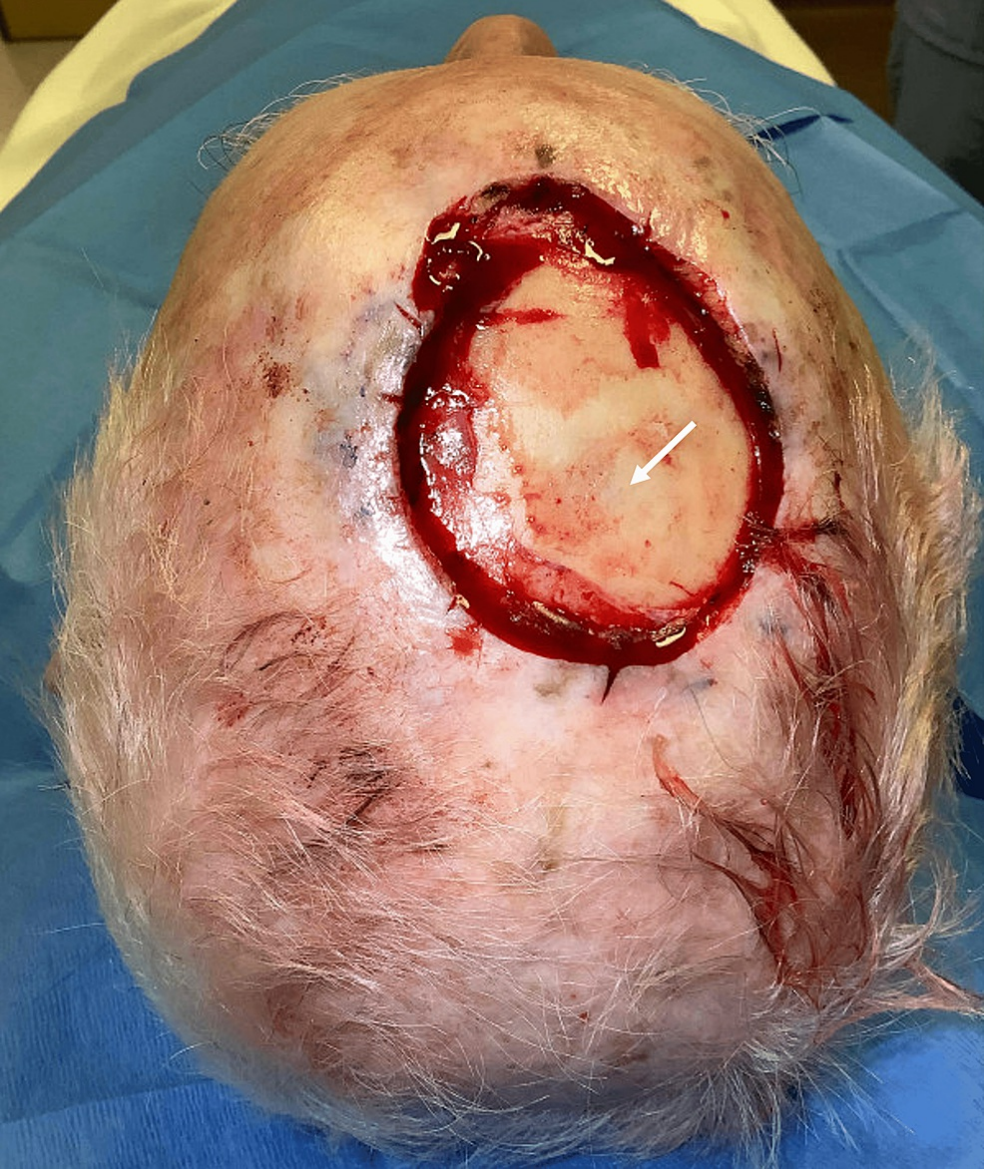
Large scalp defect measuring 7.5 x 6.0 cm with exposed calvarium (white arrow)

A 79-year-old male patient with a history of multiple prior keratinocyte carcinomas was referred to our institution for Mohs micrographic surgery of a biopsy-proven squamous cell carcinoma on the right medial frontal scalp. Tumor-free margins were obtained after three stages of Mohs micrographic surgery, with a final defect measuring 7.5 x 6.0 cm with an extension to the level of the calvarium (Figure 1). 

**Figure 2:**
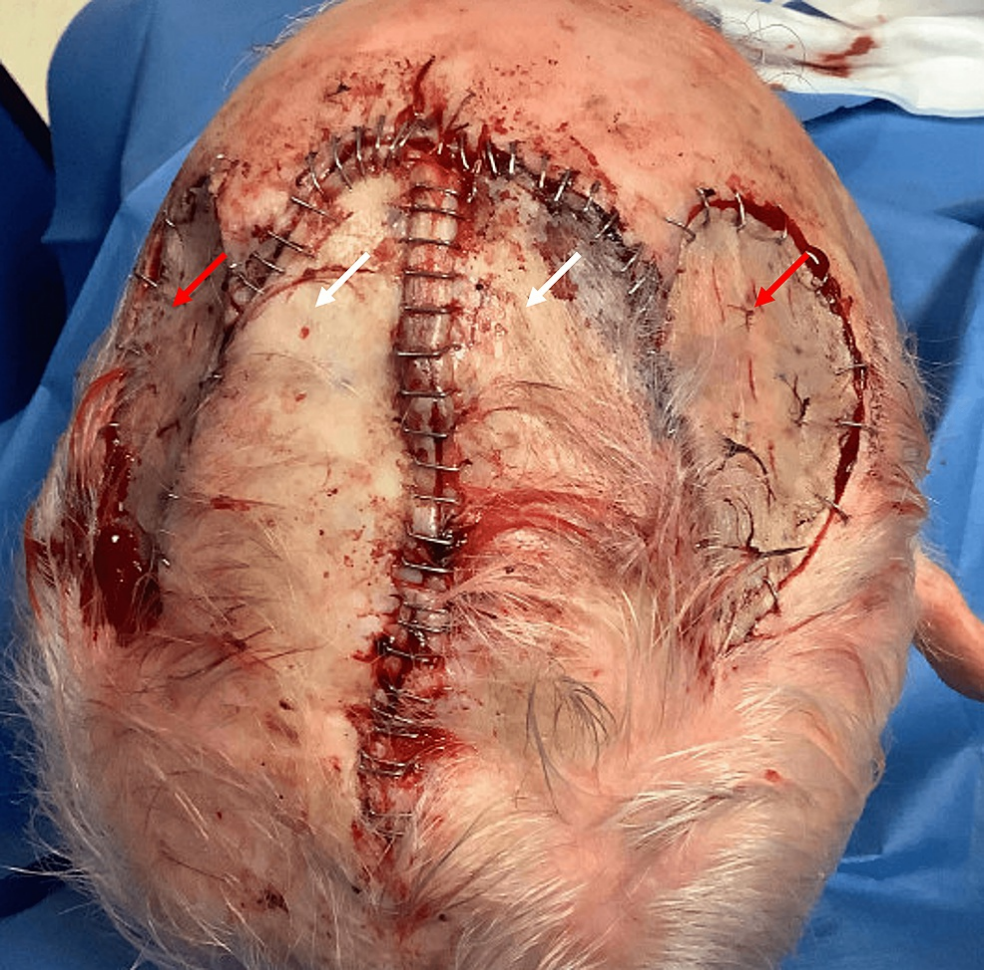
Bird's-eye view of the patient's scalp following reconstruction with bilateral transposition flaps (white arrows) and split-thickness skin grafts (red arrows) to repair the secondary defects

**Figure 3:**
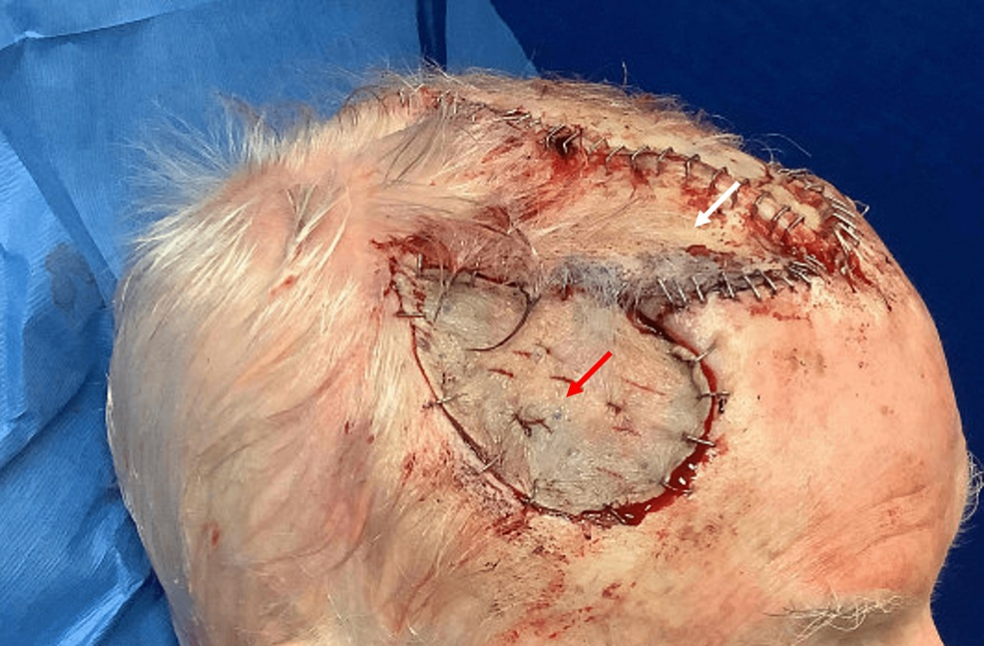
Right lateral view of patient's scalp following reconstruction bilateral transposition flap (white arrow) and split-thickness skin graft (red arrow) to repair the secondary defect

**Figure 4:**
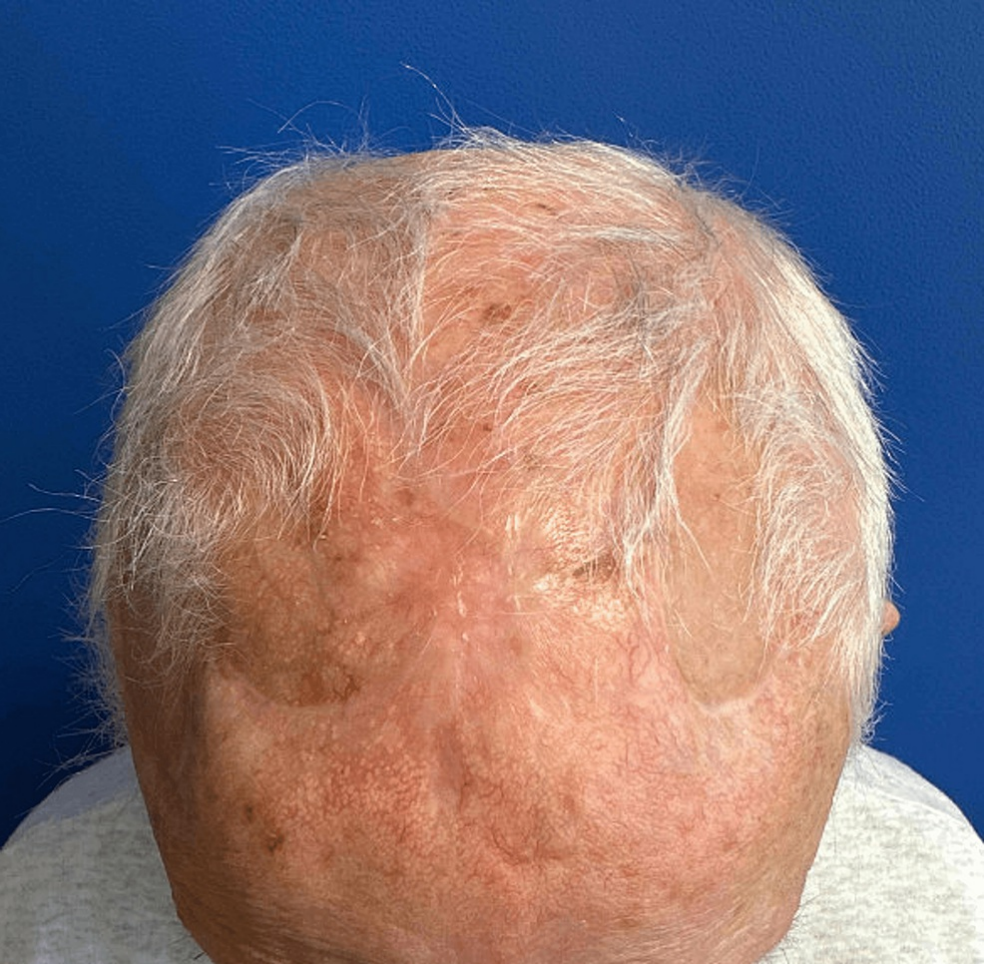
Bird's-eye view of the patient's scalp five months after repair

The decision was made to repair the defect with two laterally based transposition flaps taken from either side of the primary defect. Incisions were taken down to the periosteal plane, after which the flaps were dissected and elevated to the respective pedicle bases. The surrounding skin was widely undermined at the level of periosteum. The flaps were then transposed into the primary defect and sutured together, covering the exposed calvarium. An excess standing tissue cone was removed posteriorly from the primary defect, taking care not to compromise the flap pedicles. The epidermis was then carefully approximated using staples along the length of the flaps. The final flap size was 10.0 x 9.0 cm. At this point, two secondary defects remained following flap transposition, both with intact periosteum. The secondary defect on the right scalp measured 6.5 x 3.8 cm and the left measured 6.2 x 2.9 cm. To close the secondary defects, a split-thickness skin graft was harvested from the left lateral thigh. The graft was fenestrated, rinsed in saline, and then secured to the secondary defects using staples (Figures 2-4). Basting sutures were used to tack down the graft centrally and then a bolster dressing was placed. 

 

## Discussion

While small scalp defects following surgical treatment of cutaneous malignancies can often be repaired with linear closure or allowed to granulate, large scalp defects create a more difficult reconstructive challenge. This location can often demonstrate poor local tissue availability, limiting repair options. Extension of the defect beyond well-perfused planes such as the pericranium or fascia to the level of the calvarium further complicates the repair. In general, repair options for large, deeply extending scalp defects include healing by second intention, full or split-thickness skin grafts with or without fascial flap, advancement or transposition flap with or without the aid of tissue expanders, free-tissue transfer, or a combination of these techniques. For defects extending to the calvarium, healing by second intention or the use of skin grafts is limited due to lack of perfusion to the wound bed. Healing by second intention can be complicated by infection or bone necrosis [1]. Grafts are also less likely to survive when placed directly on the calvarium than on well-perfused planes such as the pericranium or fascia [2]. An additional drawback to this approach is a tendency towards poor aesthetic results [3]. Galeal, pericranial, and temporoparietal fascial flaps have been used to improve graft survival by providing a means of flap perfusion; however, these flaps can be difficult to perform due to the tight adherence of these scalp layers [4]. While rotation flaps with lengthy incision lines can be utilized, the availability of scalp skin is often a limiting factor. As such, utilization of flaps designed on the skin adjacent to the primary defect with subsequent repair of donor sites via STSG has been proposed as a method to improve repair outcomes [5]. While tissue expansion followed by flap repair or free-tissue transfer are feasible repair options, they are typically unable to be performed as a single operative procedure and have a higher risk of complications. 

**Figure 5:**
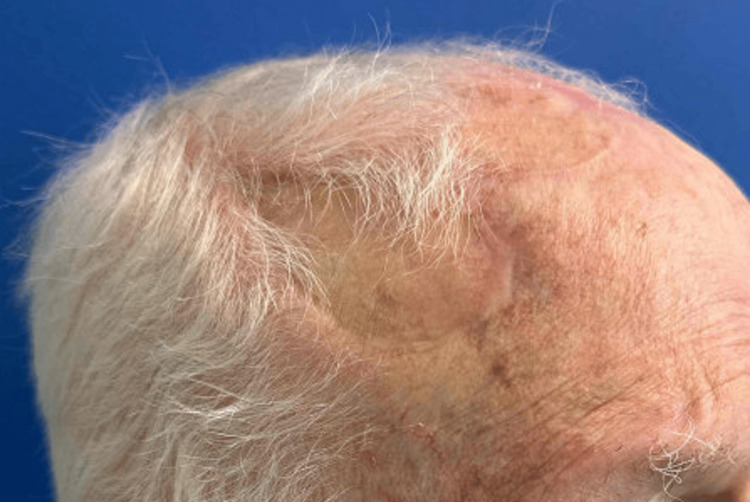
Right lateral view of patient's scalp five months after repair

Here we report a unique reconstruction of a large scalp defect using bilateral transposition flaps with repair of secondary defects utilizing STSG. This allowed same-day reconstruction in line with patient preference, in addition to flap repair of the primary defect with exposed calvarium. The patient returned three months after the procedure with an acceptable functional and aesthetic outcome (Figures 4, 5) considering the size and location of the defect. 

## Conclusions

Large scalp defects with extension to the calvarium represent a reconstructive challenge. Repair of the primary defect with a transposition flap followed by STSG to the secondary defect is a reliable method of reconstruction that allows for same-day repair with excellent functional and cosmetic outcomes. This case highlights a unique reconstruction utilizing bilateral transposition flaps followed by STSG repair of the secondary defects. 
